# Consequences of VHL Loss on Global DNA Methylome

**DOI:** 10.1038/s41598-018-21524-5

**Published:** 2018-02-20

**Authors:** Claire M. Robinson, Francois Lefebvre, Betty P. Poon, Aurelie Bousard, Xiaojun Fan, Mark Lathrop, Jorg Tost, William Y. Kim, Yasser Riazalhosseini, Michael Ohh

**Affiliations:** 1 Department of Laboratory Medicine and Pathobiology, University of Toronto, 661 University Avenue, Room 1510, M5G1M1 Toronto, Ontario Canada; 2 Department of Biochemistry, University of Toronto, 661 University Avenue, Room 1510, M5G1M1 Toronto, Ontario Canada; 3 Canadian Centre for Computational Genomics (C3G), 740 Doctor Penfield Avenue, Montreal, QC H3A 0G1 Canada; 4 Laboratory for Epigenetics & Environment, Centre National de Génotypage, CEA-Institut de Génomique, 2 rue Gaston Crémieux, 91000 Evry, France; 5 Department of Human Genetics, McGill University, 1205 Dr Penfield Avenue, Montreal, QC H3A 1B1 Canada; 6 McGill University and Genome Quebec Innovation Centre, 740 Doctor Penfield Avenue, Montreal, QC H3A 0G1 Canada; 7 Lineberger Comprehensive Cancer Center, University of North Carolina at Chapel Hill, CB 7295, Chapel Hill, North Carolina USA

## Abstract

In clear-cell renal cell carcinoma (ccRCC), loss of von Hippel-Lindau (VHL) tumour suppressor gene and reduced oxygen tension promote stabilisation of hypoxia-inducible factor (HIF) family of transcription factors, which promote changes in the expression of genes that contribute to oncogenesis. Multiple studies have demonstrated significant perturbations in DNA methylation in ccRCC via largely unclear mechanisms that modify the transcriptional output of tumour cells. Here, we show that the methylation status of the CpG dinucleotide within the consensus hypoxia-responsive element (HRE) markedly influences the binding of HIF and that the loss of VHL results in significant alterations in the DNA methylome. Surprisingly, hypoxia, which likewise promotes HIF stabilisation and activation, has relatively few effects on global DNA methylation. Gene expression analysis of ccRCC patient samples highlighted expression of a group of genes whose transcription correlated with methylation changes, including hypoxic responsive genes such as VEGF and TGF. These results suggest that the loss of VHL alters DNA methylation profile across the genome, commonly associated with and contributing to ccRCC progression.

## Introduction

Renal cancer comprises approximately 3% of all cancers and is one of the top ten leading causes of cancer related mortality in the United States^1,2^. Although the disease can be categorised into various histological subtypes, clear-cell renal cell carcinoma (ccRCC) is the most common, accounting for more than 70% of all cases. This highly aggressive, chemotherapy resistant tumour type is characterised genetically by loss or mutation of von Hippel-Lindau (*VHL*) gene, proposed to occur in up to 80% of all ccRCC^3^.

VHL status in ccRCC does not correlate with clinical outcome; however, it can be described as a classic tumour suppressor protein and has been shown to exert multiple anti-oncogenic functions^3^. Chief amongst these and the best studied to date is its role in regulation of the hypoxia-inducible factor (HIF) family of transcription factors^4,5^. HIFs are widely expressed and under normal oxygen tension become hydroxylated by a family of oxygen and α-ketoglutarate dependent enzymes called prolyl hydroxylases (PHDs). As the substrate-conferring component of the ECV (Elongins BC/Cul2/VHL) ubiquitin ligase complex, VHL binds only to the hydroxylated form of HIFα, resulting in polyubiquitylation and subsequent degradation of the HIFα subunit by the 26S proteasome^6^. Thus, an important consequence of VHL loss or mutation in ccRCC is HIF stabilisation. Stabilised HIFα dimerises with the constitutively expressed HIFβ and translocates to the nucleus where it acts to upregulate a wide variety of genes including those involved in glycolysis, angiogenesis, cell proliferation and survival. A significant proportion of altered gene expression in ccRCC is attributed to transcription mediated by the VHL-HIF axis^7^. As their name suggests HIFs are also stabilised in response to inadequate oxygen supply, or hypoxia, where they act to promote cell survival. The PHDs are oxygen dependent and their activity is inhibited in hypoxic environments. In these circumstances, HIF is not hydroxylated, evades VHL recognition and escapes degradation. Hypoxic environments are classic features of solid tumors, including ccRCC, where hyperactive, dysregulated vascular growth results in poorly oxygenated regions and subsequent HIFα stabilisation. This combination of VHL loss often alongside areas of inadequate oxygen supply gives rise to a tumour environment that is characterised by high HIFα expression at a variety of oxygen tensions ranging from normoxia to severe hypoxia.

As is the case for all transcription factors, there must be adequate access to respective binding regions of DNA in order for transcriptional activation to occur^8^. HIFα binds to hypoxia response elements (HREs) containing the consensus sequence RCGTG (where R is A or G) to upregulate gene transcription^9^. The likelihood of transcription factor binding is often determined by epigenetic factors and previous studies have demonstrated that changes to epigenetic code surrounding HREs can augment HIF mediated transcription^10,11^. Interestingly, regulation in the opposite direction appears to also occur, whereby HIF stabilisation can elicit changes in the epigenetic code^12,13^. HRE sequences are of particular interest in relation to DNA methylation as there is a CpG dinucleotide within the consensus sequence. CpG dinucleotides can become epigenetically altered via DNA methylation, whereby the 5^th^ carbon of the cytosine residue gains a methyl group to become 5-methylcytosine (5MeC). The complexities of DNA methylation continue to be discovered^14^. However, in general terms methylation is associated with chromatin condensation and transcriptional repression, whilst loss of DNA methylation is associated with a loosening of chromatin and at gene promoters can result in upregulated gene expression^15^.

Global hypomethylation alongside promoter specific hypermethylation events are common features of most cancers. The Cancer Genome Atlas Research Network along with studies on smaller patient cohorts reported these as features of ccRCC patient samples^16^. Hypomethylation is thought to contribute to genome instability while promoter specific hypermethylation contributes to disease progression via silencing of anti-oncogenic genes^17^. Despite the common occurrence of these significant changes in the DNA methylome, little is known about the mechanisms in renal cancer cells that cause such distinct changes in DNA methylation and the repercussions of such changes to HIF mediated gene expression. This study was undertaken in order to investigate these questions in relation to ccRCC. Given that HIF stabilisation as a consequence of VHL loss and/or hypoxia are important events in the pathogenesis of ccRCC, alongside the fact that HIF has previously been implicated in modifying the epigenome, we asked whether these two events impact the DNA methylome of ccRCC.

## Results

### CpG methylation within HRE prevents HIF binding and subsequent transcription

**Figure 1:**
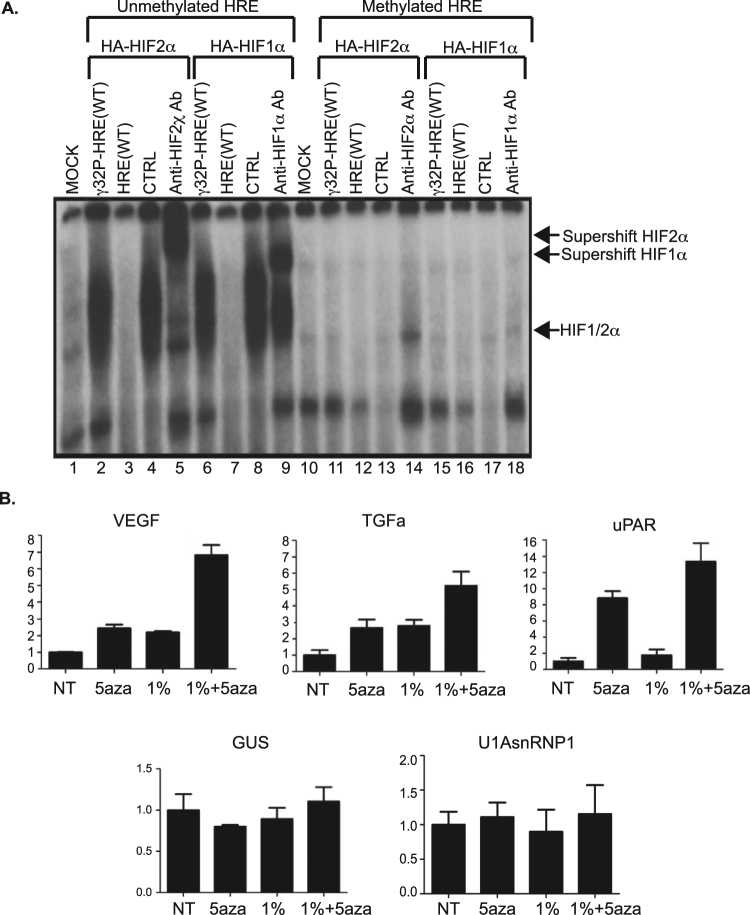
Manipulation of DNA methylation at HRE sequences alters HIF binding. (**A**) The Hypoxia Response Element (HRE) contains a CpG site that can be methylated which prevents HIF1 and HIF2 binding *in vitro*. EMSA of *in vitro*–translated HIF1 and HIF2 binding to 32P-labelled unmethylated and methylated HRE probes. Competition with 250X molar excess of unlabelled/unmethylated HRE probe (lanes 3, 7,12, 16) or unlabelled control HRE-free probe (lanes 4, 8, 13, 17). HIF1 and HIF2 complexes bound to unmethylated HRE or methylated HRE supershifted with anti-HIF1α (lanes 9 and 18) and anti-HIF2α (lanes 5 and 14). (**B**) RCC4-VHL cells were grown in normoxia (NT) or treated with 25 µM decitabine (5aza) or grown in hypoxia (1%) for 48 hrs and with the addition of 25 µM decitabine (1% + 5aza). QPCR was performed using primers specific  to *VEGF, uPAR, TGFα, GUS, U1AsnRNP1*. Samples were normalised to relative expression of the housekeeping gene, *ACTIN*.

HIF binds to the consensus HRE sequence (5′-(A/G)CGTG-3′) within promoters and enhancers to upregulate hypoxia-inducible gene transcription. The number of direct HIF target genes is intriguingly and significantly less than the number of HRE sequences found in the genome potentially available to HIF. Epigenetic modification is a potential mechanism that may prevent HIF binding to inappropriate sites. Considering that the HRE sequence contains a CpG dinucleotide capable of undergoing changes in DNA methylation, we asked whether CpG methylation at HRE would have an impact on HIF binding. The radiolabelled *VEGF* HRE probes containing either synthetically methylated or unmethylated CpG dinucleotide were analysed for binding to HIF1α and HIF2α via electrophoretic mobility shift assay (EMSA) (Fig. 1A). Both HIF1α and HIF2α bound robustly to radiolabelled unmethylated HRE probes, which were abolished by increasing amounts of competing unlabelled unmethylated HRE probes (Fig. 1A, lanes 2–3 and lanes 6–7). In contrast, neither HIF1α nor HIF2α was capable of binding radiolabelled methylated probe, even in the absence of competing unlabelled methylated probe (Fig. 1A, lanes 11 and 15). These results demonstrate that the affinities of both HIF1α and HIF2α for HRE sequence are markedly reduced when the CpG within HRE is methylated.

Consequently, the presence of methylation at HREs would have a marked impact on HIF mediated gene expression. Previous studies have also alluded to this and HIF responsive genes such as *CAIX* and *EPO* are both repressed if their respective HREs or areas in the vicinity of the HRE are methylated^11,18^. Considering the EMSA results, we next asked whether removal of CpG methylation would affect HIF mediated gene expression of known HIF responsive genes. For these studies we used human ccRCC cells stably expressing HA-tagged wild-type VHL (RCC4-VHL). Despite the fact that VHL degrades HIFα via ubiquitin-proteasome system in an oxygen-dependent manner, RCC4-VHL cells express low levels of HIFα in normoxic conditions whereas in hypoxia HIFα is dramatically stabilised. These cells were treated with the DNA methylation inhibitor 5-aza-2-deoxycytidine (decitabine) in normoxia and in hypoxia. Gene expression levels of classic hypoxic responsive genes (*VEGF*, *uPAR* and *TGFα*), alongside 2 genes that do not contain HREs (*GUS* and *U1AsnRNP1*), were quantified using qPCR (Fig. 1B). Interestingly hypoxic responsive gene expression was enhanced in both normoxia and hypoxia when cells were treated with decitabine whereas expression of genes without HREs remained unchanged. The increased expression in normoxia upon decitabine treatment was similar to the expression induced by growing cells in hypoxia, except for uPAR. While demethylation of tissue-specific enhancers or epigenetically silenced trans-acting transcription factors could impact transcription of HRE-containing genes, it is equally likely that that regardless of oxygen tension, a certain degree of methylation may exist at or near many of these HREs, which acts to dampen HIF mediated transcription and that removal of DNA methylation enhances HIF mediated transcription.

### Loss of VHL results in significant changes to the DNA methylome

**Figure 2:**
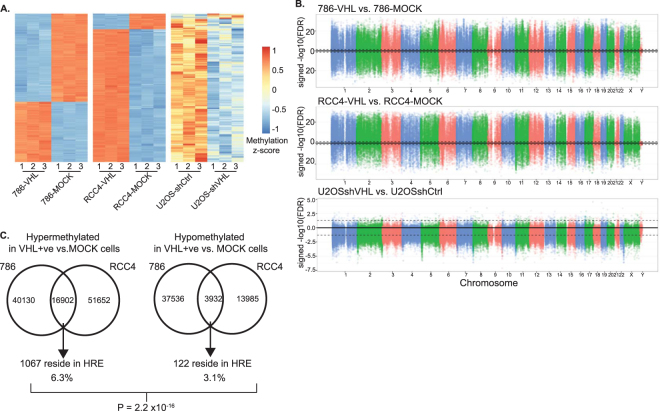
Loss of VHL is associated with significant changes in 5MeC in ccRCC cells. (**A**) Heatmap clustering of DNA methylation levels (M-values) in parental and VHL-modulated derivatives of 786 (left) RCC4 (middle) and U2OS (right) cell lines. Numbers beneath the heatmaps indicates the biological replicates. M-values corresponding to the methylation levels of the 10000 most significantly differentially methylated loci in experiments were used to generate the heatmaps (**B**) Manhattan plots show genome-wide distribution of DNA methylation in 786 (top), RCC4 (middle) U2OS (bottom) cell lines. X-axis represents chromosomes and Y-axis FDR values for differential methylation analysis. Dashed lines represent FDR = 0.01 (**C**) Numbers of CpG loci exhibiting significant differential DNA methylation between parental (mock) and VHL-reconstituted (VHL+) derivatives of two ccRCC cell lines 786 and RCC4 are shown. Among loci that show the same direction of change in methylation upon VHL expression in both cells lines, the overlap with those residing in HRE features are depicted.

While pharmacological manipulation of DNA methylation in our cell system can alter HIF mediated transcription, we questioned whether changes at CpG sites occur in situations where HIF is known to be constitutively active and therefore capable of mediating transcription. Significant changes in DNA methylation are evident when normal renal tissue is compared to patient renal tumours^16^. We hypothesised that VHL loss, the most commonly occurring genetic alteration in ccRCC and accompanied HIF stabilisation, may contribute to the altered epigenome in ccRCC. Under these circumstances we proposed that methylation changes at HREs could effectively heighten and/or alter the HIF mediated response. In order to investigate DNA methylation differences, we employed a genome-wide approach by using Illumina human methylation 450 K arrays. These arrays offer valuable insight into the degree of methylation at a wide variety of CpG residues at various positions across the genome. We therefore chose to compare genome-wide status of 5MeC levels in ccRCC cells lacking VHL versus cells with VHL stably reconstituted by a differential DNA methylation analysis. This analysis uncovered significant differences between the DNA methylation profiles of RCC4-VHL and RCC4-mock cells with 68,554 hypermethylated and 17,917 hypomethylated loci (FDR <0.05 Benjamini-Hochberg corrected with an absolute difference of M-value greater than 1, Supplementary Table 1; Fig. 2A) in the former. 5MeC levels were investigated in two other cell types with or without VHL (786-mock versus 786-VHL and U2OSshSCR versus U2OSshVHL). 786 cells, much like RCC4 cells are human ccRCC cells although unlike RCC4 cells, these cells are HIF1α negative and only express HIF2α. Similar to our results from RCC4 cells, we identified a large number of CpG sites with significant differential DNA methylation patterns between 786-mock and 786-VHL cells; 57,122 hypermethylated and 41,163 hypomethylated in 786-VHL cells relative to 786-mock cells (Supplementary Table 2; Fig. 2A). Human osteosarcoma U2OS cell lines stably expressing non-targeting shRNA or shRNA against endogenous VHL were generated and 5MeC levels were measured using the same arrays. Differential methylation analysis identified 91 and 2523 loci as being significantly hyper- and hypomethylated, respectively, following VHL knockdown (Supplementary Table 3; Fig. 2A). The differentially methylated loci were identified across the genome in all three cell lines (Fig. 2B) highlighting that VHL status has a global effect on DNA methylome.

Given the significant changes in DNA methylation between VHL+ve and isogenically matched VHL−ve cells, we next investigated what CpG sites were being altered in these cells. We focused our analysis on RCC4 and 786 cells as both of these cell lines are derived from ccRCC harbouring a loss of endogenous VHL and thus are more relevant for studying functional consequences of VHL loss in ccRCC pathogenesis. Our preceding analyses of 786 and RCC4 cells uncovered many thousands of sites that showed significant changes in their methylation upon re-expression of VHL (Supplementary Tables 1 and 2). When combining data of 786 and RCC4 cell lines, we identified 20,834 loci that exhibited similar changes in these two cell lines upon ectopic expression of VHL; 16,902 with hyper- and 3,932 with hypomethylation. To investigate the potential contribution of DNA methylation of these loci to the hypoxia-mediated transcription, we examined the coincidence of these loci with canonical HRE and observed 2-fold enrichment of hypermethylated loci in HREs (p = 2.2 × 10^−16^, Fisher’s exact test) compared to hypomethylated loci (Fig. 2C). These results suggest that VHL-dependent DNA hypermethylation may affect HIF-mediated gene expression by altering epigenetic status of HREs, thereby modulating HIF binding to HREs.

### Hypoxia has minimal effects on global 5MeC that is likely governed by repression of DNMT1

**Figure 3:**
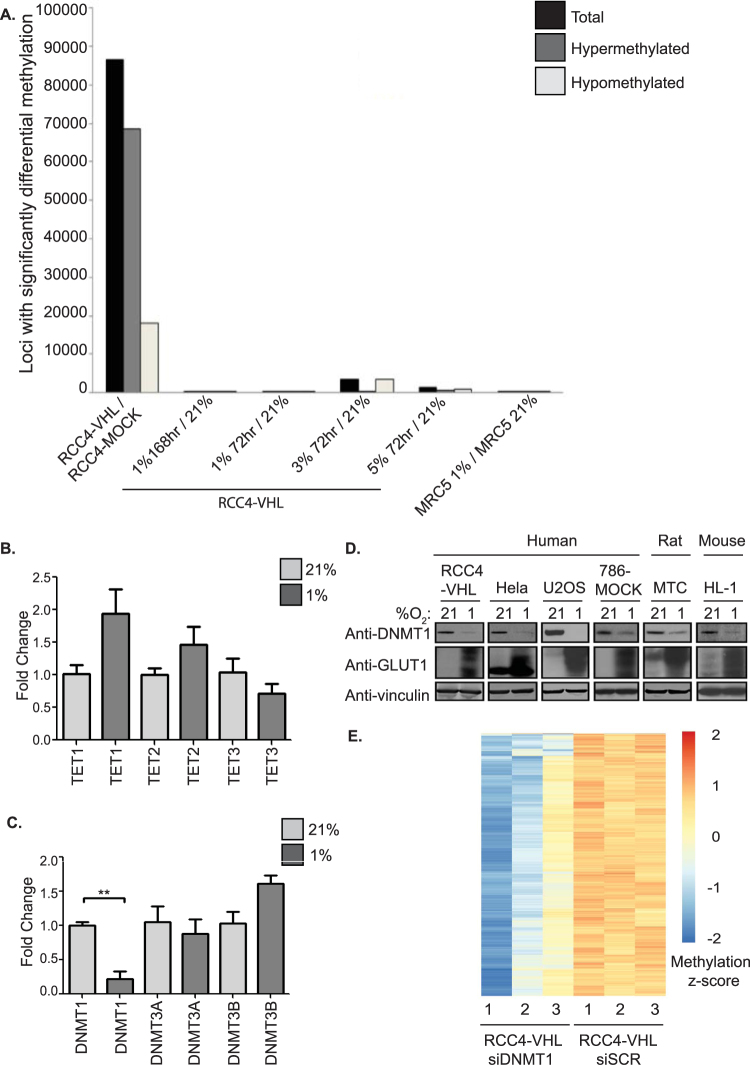
Hypoxia causes minor changes in DNA methylation via DNMT1 repression. (**A**) Number of differentially methylated loci (FDR <0.05 and M >0.2) between the two conditions is shown for each experiment. (**B**) mRNA was extracted from RCC4-VHL cells grown in normoxia or hypoxia (1% O_2_, 48 hrs). QPCR was performed using primers specific to *TET1*, *TET2* and *TET3* or to (**C**) DNMT1, DNMT3A and DNMT3B. In all QPCRs, samples were normalised to relative expression of the housekeeping gene, *U1AsnRNP1* (**D**) Multiple cell types were grown in 1% O_2_ for 48 hrs, protein was extracted and expression of DNMT1 was quantified using western blot. Vinculin was used as the loading control. Immunoblots depicted are a single representative of multiple independent experiments. (**E**) Heatmap clustering of DNA methylation levels (M-values) in RCC4-VHL cells treated with siSCR or siDNMT1. Numbers beneath the heatmaps indicates the biological replicates. M-values corresponding to the methylation levels of the 10000 most significantly differentially methylated loci in experiments were used to generate the heatmap.

As we recorded dramatic changes in DNA methylation between cells with and without VHL, we next performed similar experimental procedure to investigate the effect of hypoxia on DNA methylation patterns. Hypoxia occurs in a variety of situations in both health and disease, including cancer. It has previously been shown that vast changes in the chromatin signature are required to elicit a complete hypoxic response^10,19^; we therefore hypothesised that this process would likely involve changes in DNA methylation marks, some of which may occur at putative and/or recognised HREs. Surprisingly the results from these experiments revealed striking similarities between the DNA methylomes of RCC4-VHL cells grown for 72 hours in 1% O_2_ and 21% O_2_, (Fig. 3A); there were only 7 loci showing significant differential methylation between them. Considering this lack of change in 5MeC between normoxic and hypoxic cells, we questioned whether the results that were recorded in RCC4-VHL cells were simply an anomaly specific to that cell line. In this regard, we performed the same hypoxic experiment using human fetal lung fibroblast cell line, MRC5. Consistent with the results obtained in the ccRCC cell line, we identified only 193 (1 hyper- and 192 hypomethylated) loci with differential methylation levels between MRC5 cells grown in hypoxic and normoxic conditions. Initially we considered that perhaps 72 hrs was not sufficient time for the 5MeC levels to significantly change. To investigate this possibility, RCC4-VHL cells were grown in 1% O_2_ for 168 hours (7 days) and DNA methylation was measured. However, no significant changes between normoxic and chronically hypoxic cells were apparent (total of 33 CpG sites exhibited differential methylation; Fig. 3A). Given that TET enzymes, as well as other epigenetic modifiers, are oxygen dependent, we next hypothesised that perhaps hypoxic cells required a higher level of oxygen in order to maintain activity of all DNA methylation machinery. Hence, ccRCC cells were additionally grown in 3% and 5% O_2_. In these experimental conditions HIFα remains stabilised. However, our genome-wide analysis revealed slight to moderate changes between normoxic and hypoxic cells grown in these O_2_ levels (3,384 and 1,231 differentially methylated loci when normoxic condition was compared to 3% and 5% O_2_, respectively, Fig. 3A; Supplementary Tables 4 and 5). These results suggest that hypoxia does not have a profound effect on 5MeC status while loss of VHL in the same cell type promotes dramatic changes in global DNA methylation (Fig. 2A).

QPCR analysis of the DNMTs as well as expression of the TETs were analysed in normoxic and hypoxic RCC4-VHL cells (Fig. 3B–C). At an mRNA level, DNMT1 transcription was significantly repressed in hypoxic cells (Fig. 3C). Protein expression analysis revealed that DNMT1 expression was markedly attenuated during 48-hour growth in hypoxia in a wide variety of cells of human, rat and mouse origin (Fig. 3D). DNA methylation can be lost passively via suppression of DNA methyltransferases. Therefore it is likely that the decrease in DNMT1 mediates many of the changes seen in hypoxic cells. To address this notion, RCC4-VHL cells were transfected with non-targeting scrambled siRNA (siSCR) or DNMT1-targeting siRNA (siDNMT1) and simultaneously grown in normoxia. Illumina 450k arrays were used to interrogate changes in DNA methylation. There were slight changes in DNA methylation (999 loci) upon DNMT1 knockdown in RCC4-VHL cells maintained in normoxia (Fig. 3E), which was relatively similar in number to the changes recorded in hypoxic cells. Although the numbers of CpG sites were similar, 2% of these sites were the same in hypoxic cells and siDNMT1 cells. While the results suggest that the changes in DNMT1 level in hypoxia may account for the modest loss of DNA methylation recorded in these cells, the results also indicate that the location of these changes appear to be more random.

### Changes in 5MeC at various regions in primary ccRCC include HIF target genes and putative HREs

**Figure 4:**
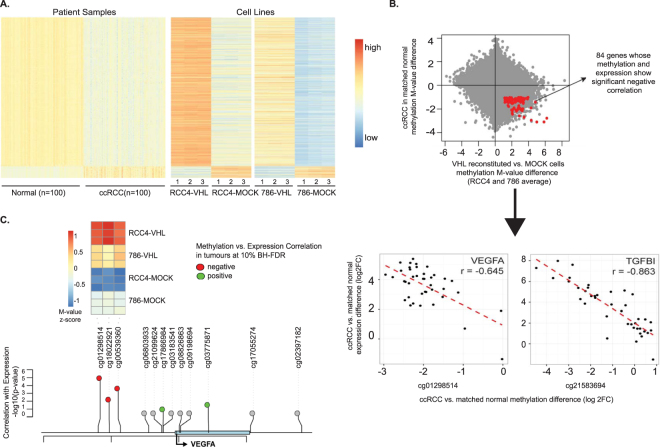
Changes in DNA methylation is evident in tumour samples with VHL loss. (**A**) Loci showing coordinate methylation changes between patient samples and VHL +/− ccRCC cell lines. Same loci ordering across panels is shown. (**B**) Loci that are hypomethylated in ccRCC tumors and gain methylation upon VHL expression in ccRCC cell lines are highlighted with red color. Correlation analysis between methylation changes (tumor/normal) of these genes and mRNA changes (tumor/normal) of genes associated with them identified 84 genes with significant inverse correlation. Correlation results for VEGFA and TGFBI are shown as examples. (**C**) Cluster analysis demonstrates a correlation between methylation at VEGF related CpGs and VEGF expression upon VHL loss.

We next compared the results from methylation profiling of ccRCC cell lines to the methylome of human primary ccRCC specimens, which are characterised by inactivation of VHL due to somatic mutations or promoter hypermethylation. We used differential methylation data between ccRCC samples and matched normal kidney tissue from 100 individuals, which were profiled with the same arrays. This analysis showed that VHL re-expression in cell lines can change methylation of 2197 out of 34598 loci with ccRCC-associated methylation to the patterns observed in normal kidney tissue, suggesting that VHL loss contributes to the abnormal methylation of 6.3% of affected loci. It is notable that this fraction represents CpG loci with high-confidence methylation changes observed recurrently across multiple tumors. Therefore, the extent of VHL-dependent DNA methylation changes across the genome may well be much broader. However, among 18,191 CpGs that were hypomethylated in ccRCC as compared to patient-matched normal kidney tissues, 2,037 loci (11.2%) become hypermethylated upon ectopic expression of VHL in ccRCC cell lines. In contrast, among 16,407 CpG sites that exhibited hypermethylation in ccRCC tumors, only 160 loci (~1%) showed hypomethylation in VHL-reconstituted cell lines. These findings suggest that, in regards to epigenetic aberrations, VHL deficiency contributes predominantly to the loss of DNA methylation in ccRCC tumors (Fig. 4A, Supplementary Table 6)

We further investigated possible correlation between abnormal methylation of these loci and cancer associated expression of the genes associated with them. Given the well-known negative regulation of gene expression by DNA methylation, we focused our analysis on genes that were associated with hypomethylated loci and were upregulated in ccRCC tumours compared to normal kidney samples. Our analysis identified 83 genes that displayed significant negative correlation between methylation status of their associated CpG loci and expression of their mRNA across sample pairs (Pearson correlation <−0.3, p < 0.05; Supplementary Table 7). Pathway analysis of these genes showed enrichment in various pathways including many immune system and cell signalling related pathways. Furthermore, several genes implicated in ccRCC development or progression were distinctly altered in both tumour samples and cells. Intriguingly, and in support of our previous findings, these genes included HIF responsive genes such as VEGFA, TGFBI^20^. While the loss of 5MeC did not occur in VEGF HRE, significant alterations did take place in the promoter (Fig. 4B). In addition many other genes with putative HREs were also affected, including TRIB3, INSR and CD48. Similar analysis with genes associated with loci that showed hypomethylatation upon VHL expression in cell lines and hypermethylatation in ccRCC tumors returned only 3 genes whose expression showed negative correlation to the CpG methylation; HOXB8, FAM89A and PPP2R2D, of which none were among the HIF-target genes.

According to the role of DNA methylation of promoter CpG islands on the regulation of gene expression, we also investigated associations between changes of DNA methylation of promoter CpG islands and alterations in expression of nearby genes in ccRCC, and the potential involvement of VHL. To this end, we first identified cluster of CpGs, which are annotated to the same promoter CpG islands, and represented similar changes (hyper or hypomethylation) in ccRCC compared to matched normal samples, and then examined association between weighted average methylation value of these cluster and expression of the associated gene (see Methods for details). This analysis resulted in 42 genes whose expression changes showed a negative correlation with changes of DNA methylation of their promoter CpG clusters, which consisted of minimum of three CpGs (Supplementary Table 8). We observed that methylation of clusters associated with fourteen of these 42 genes is changed upon expression of VHL in either 786-O or RCC-4 cell lines (Supplementary Table 8). These include VEGFA, which was also identified by our single CpG analysis.

## Discussion

VHL is a multifunctional protein. To date the best described function of VHL is its role as the substrate recognition subunit of the ECV complex that participates in the degradation of HIF. HIF independent functions for VHL have also been reported including its involvement in the degradation of other target proteins^21,22^, as well as suggested roles independent of its E3 ubiquitin ligase function^23^. Here we report a potential role for VHL in the regulation of the DNA methylation profile of cells. The solitary loss of VHL resulted in dramatic changes in DNA methylation in different cell types. Despite the fact that ectopic VHL expression in the cell lines tested could result in promoting supraphysiological effects on the DNA methylome, many of the changes we recorded in the cell lines were evident in ccRCC patient samples where loss or mutation of VHL is the most common and earliest genetic perturbation.

In contrast to the results observed upon manipulation of VHL, this study demonstrates that in hypoxia where HIF is also stabilised, there is relatively little change in the global DNA methylome. Given that in both situations HIF is activated, it is somewhat surprising that there is such a vast difference in the methylome between these conditions, i.e., hypoxia versus VHL loss. This result is particularly intruiging as previous investigations have reported an association between HIF stabilisation and promotion of epigenetic modifications including DNA methylation. Many epigenetic enzymes contain putative HREs and have been proposed to be HIF target genes. These epigenetic modifiers, like the PHDs, also require oxygen for their enzymatic function. When TET enzymes are active, they oxidize conversion of 5mC to 5hmC which through a series of TET mediated reactions is converted to 5fC or 5caC. 5MeC will eventually be returned to cytosine, the net result being demethylation. Although they are robustly expressed in the ccRCC cells used in this study, TET mRNA expression was unchanged in hypoxic RCC4-VHL cells. This finding is in support of a recent study that quantified TET mRNA expression in a variety of hypoxic cells. While mRNA expression varied, activity of these enzymes was inhibited providing an effective mechanism to incur hypermethylation events^24^. Other studies have also interrogated the relationship between hypoxia and TETs and demonstrated that HIF1α mediated induction of TET1 promotes increased global 5hMeC in neuroblastoma cells but not in other cell types tested^25^. Interestingly in this study, despite manipulation of hypoxic time point to 7 days as well as the degree of hypoxia, there was very little effect on global 5MeC, suggesting the unlikely suppression of TET activity in this system. In fact the small degree of demethylation in hypoxia could be accounted for by the loss of DNMT1. Loss of DNMT1 results in passive removal of 5MeC during cell replication although other reports implicate that loss of DNMT1 may also have consequences independent of its catalytic activity^26^.

While a reduction in DNMT1 may account for most of the changes evident in the methylome in hypoxia, its loss does not provide a dynamic enough pathway to account for the vast changes evident in ccRCC cells lacking VHL. Loss of VHL leads to significant hyper and hypomethylation changes. Given previous reports in the literature regarding HIF mediated regulation of epigenetic machinery it is possible that altered expression of the enzymes that regulate DNA methylation may occur in a HIF mediated fashion upon VHL loss. However, changes exclusive to DNA methylation enzymes may not account for all the modifications in 5MeC upon VHL loss. It is also conceivable that other yet-identified or -confirmed mechanisms contribute to the substantial changes recorded throughout the DNA methylome. For example, many ccRCC tumors harbour mutations, predominantly of epigenetic regulators such as PBRM1 and SETD2^16,27^. There is growing evidence supporting the influence and interplay of histone modifications on DNA methylation. Recently it has been demonstrated that SETD2 inactivation results in global redistribution of 5MeC in renal cells^28^. These are likely contributory factors to changes in DNA methylation, which alongside VHL may account for the diverse alterations evident in ccRCC samples. Although the precise mechanism(s) by which VHL regulates the epigenome remains an outstanding question, it is conceivable that VHL itself directly influences chromatin organisation. In support of such notion, VHL was observed to bind to heterochromatin associated protein HP1^29^. Interestingly, this interaction was not related to the degradation function of VHL instead speculating that HP1 recruits VHL to chromatin. Little is known about the functional significance of the VHL-HP1 interaction, although it is tempting to speculate that perhaps such an interaction assist in maintaining repressive chromatin structure where high levels of DNA methylation also exist.

The findings that VHL loss and hypoxia impact the DNA methylation profiles of cells differently may have very interesting implications for the development and progression of ccRCC. It is widely acknowledged that loss or mutation of VHL occurs early in tumourigenesis, while hypoxic regions of tumours are associated with later disease stages when avascular or dysregulated angiogenesis, primarily mediated by VEGF, are commonplace. Based on the observations of this study it is plausible that VHL loss early in disease progression, at a time when the tissue is well oxygenated, may significantly alter the DNA methylome. These changes could include removal of methylation at gene promoters, including HIF binding sites. As a consequence cancer cells may be capable of exaggerated expression of HIF target genes such as VEGF and TGF, as well as others. Meanwhile in hypoxia, although expression of HIF target genes occurs, our results would suggest that the amplitude is capped as DNA methylation remains and prevents an exaggerated expression capacity.

Taken together, we demonstrate that loss of VHL dramatically impacts the DNA methylation landscape of renal cancer cells while in contrast there are relatively few changes to 5MeC in hypoxia. These findings are in support of previous studies that have implicated an association between VHL loss and epigenetic modifications that promote cancer progression^30,31^. The results support the notion that VHL influences DNA methylation early in disease and likely accounts for a significant proportion of the perturbations that are evident in the DNA methylome of tumours.

## Materials and Methods

### Patient samples

Patients undergoing nephrectomy for suspected renal cancer during the period December 2008 to March 2011 at St James’s University Hospital in Leeds, UK; University Hospital Motol, Prague, Czech Republic; Masaryk Memorial Cancer Institute, Brno, Czech Republic; Th. Burghele Hospital, Bucharest, Romania; and N. N. Blokhin Cancer Research Centre, Moscow, Russia, were recruited to the study after informed consent was obtained. Recruitment in Central and Eastern Europe was coordinated by the International Agency for Research on Cancer. Ethical approvals were obtained from the Leeds (East) Local Research Ethics Committee, the International Agency for Research on Cancer Ethics Committee, as well as from local ethics committee for recruiting centres in Czech Republic, Romania and Russia. All sampling and clinical data collection was undertaken according to predefined standard operating procedures following guidelines from the International Cancer Genome Consortium. Further information about the sampling procedure, isolation of nucleic acids as well as genome and transcriptome sequencing have been described in our previous study^32^. We included DNA methylation data of ccRCC and patient-matched normal kidney tissue from 100 patients in our analysis. For correlation analysis between changes of DNA methylation and gene expression levels we focused our analysis on tumor and normal samples pairs of 40 out of the 100 patients for whom both DNA methylation and gene expression data were available.

### Cell culture and reagents

HEK293 embryonic kidney, RCC4-O(*VHL*^−^*/*^−^) and 786-O (*VHL*^−^*/*^−^; *HIF1α*^*−/−*^), U2OS, MRC5, MTC and HL-1 cells were all obtained from the American Type Culture Collection and maintained in DMEM (Gibco) supplemented with 10% (vol/vol) heat-inactivated FBS (Sigma) at 37 °C in a humidified 5% (vol/vol) CO_2_ atmosphere. RCC4 and 786 ccRCC subclones stably expressing HA-VHL (RCC4-VHL, 786-VHL) or empty plasmid (RCC4-MOCK, 786-VHL) were generated as described previously^5^. These cells were maintained in DMEM (Gibco) supplemented with 10% (vol/vol) heat-inactivated FBS (Sigma) and 1 μg/ml G418. For hypoxia treatment, cells were maintained at 1%, 3% or 5% O_2_ for the indicated times in a humidified 5% (vol/vol) CO_2_ ThermoForma hypoxia incubator at 37 °C.

### Generation of U2OS-shScr and -shVHL cells

pGIPZ shVHL and non-silencing control (shScr) plasmids were obtained from Open Biosystems (Huntsville, AL, USA). HEK293T cells were co-transfected with pMDG1.vsvg and psPAX2 (a gift from Dr Linda Penn, Ontario Cancer Institute, Toronto, ON, Canada). Viral supernatant was collected 2 days after transfection, passed through a 0.45-μm filter. U2OS cells were infected with the viral supernatant and incubated at 37  °C for 24 h. Polyclonal populations of cells stably expressing shVHL and shScr were selected with 2 μg/ml puromycin.

### Chemicals

5-aza-2′-deoxycytidine (decitabine) was purchased from Sigma. Cells were treated with decitabine for 48 hours at the concentrations indicated in the figures/figure legends.

### siRNA-mediated knockdown

ON-TARGETplus SMARTpool siRNA targeted to DNMT1 (Dharmacon, Austin, TX) and siGENOME RISC-Free Control siRNA (Dharmacon, Austin, TX) were used. RCC4-VHL cells grown on 10 cm tissue culture plates were transfected with control or DNMT1 siRNA at a final concentration of 200 nM using Oligofectamine as per manufactures instructions.

### Antibodies

Anti-HA antibody was obtained from Santa Cruz Biotechnology. Anti-HIF1α (610958) was obtained from BD Transduction Laboratories. Anti–HIF2α was from Novus Biologicals (NB100-122). Anti-glucose transporter type 1 (GLUT1) antibody was obtained from Abcam. Anti-DNMT1 (CST 5119 S) was purchased from Cell Signalling Technology. Anti- vinculin (V9264) was purchased from Sigma.

### Immunoblotting

Cells were lysed in EBC buffer and supplemented with protease inhibitors (Roche). Samples were denatured in sample buffer, and resolved by SDS-PAGE. Proteins were electrotransferred onto polyvinylidene difluoride (PVDF) membrane (Bio-Rad), blocked, and probed with the antibodies decribed above.

### Electrophoretic mobility shift assay

EMSA was performed as described previously (13). Briefly, methylated HRE (5′-GGCCAA-MeC-GTCA-MeC-GTGC-MeC-GCCCCATC-3′) and unmethylated HRE oligos (same but without methylation) were labelled with 50 μCi of γ-[32 P]-ATP, 10x PNK buffer(New England Biolabs, Beverly, MA), 1 mM spermidine (Sigma-Aldrich, Oakville, ON), and polynucleotide kinase (New England Biolabs) followed by purification using Illustra ProbeQuant G-50 Micro column (GE Healthcare). The probe was annealed at 95 °C for 5 min and cooled to room temperature. TNT T7 quick-coupled (Promega) *in vitro* translated HA-HIF1α, HA-HIF2α, pcDNA3 and ARNT (HIFβ) were combined with 10x binding buffer (40% glycerol, 50 mM Tris pH 7.5, 100 mM KCl, 10 mM DTT, 2 mg/ml BSA, and 0.2% Triton x100), 300 ng of salmon testes DNA (Sigma-Aldrich) and 100,000 cpm of labelled probe to a total volume of 25 μl. For competition, 250 molar excess of unlabelled HRE probe was incubated with the reaction mixture. The reaction was incubated at room temperature for 15 min. Supershift was performed using 1 μg of anti-HIF1α or anti-HA antibodies and incubating the reaction for an additional 15 min. Complexes were resolved on a non-denaturing 5% polyacrylamide gel and visualised by autoradiography.

### Quantitative polymerase chain reaction

RNA was isolated using RNease Mini kit (Qiagen, Mississauga, ON) and treated with DNase I (Ambion, Austin, TX) to remove residual DNA. RNA concentration was determined by spectrophotometry, and 2.5 or 5 µg of total RNA from each sample was reverse transcribed by oligo dT priming. The cDNA samples were used in real‐time PCR experiments. Ssso Advanvanced SYBR Green Supermix (BioRad) was used for qPCR reactions according to the manufacturer’s instructions. Amplification conditions were as follows:

95 °C (3 min), 40 cycles of 95 °C (10 s), 65 °C (15 s), 72 °C (20 s), and 95 °C (15 s). QPCR was performed using the ABI Prism 7900HT Sequence Detection System (Applied Biosystems). VEGF (5′-CTCTCTCCCTCATCGGTGACA-3′and 5′-GGAGGGCAGAGCTGAGTGTTAG-3′), TGFα (5′-TCTTCCAGGGTGCGGAGAT-3′ and 5′-CGGCTTTGTGGCTTCAATG-3′), uPAR (5′-GCCGGGCTGTCACCTATTC-3′ and 5′-GCCCCTCTCACAGCTCATGT-3′), GUS (5′-TTCCCTCCAGCTTCAATGACA-3′ and 5′-TAGGAATGGGCACTGCCAAT-3′), β-Actin (5′-AAAGCCACCCCACTTCTCTCTAA-3′ and 5′-ACCTCCCCTGTGTGGACTTG-3′) DNMT1 (5′-TCCTTCTGGCCTCAACTTGAA-3′ and 5′-TCCCCGGCCTCGTCATA-3′), DNMT3A (5′- CGGACATGTGGGTGGAACCTG-3′ and 5′- GCACTTCTGCCGCACCTCGT-3′), DNMT3B (5′-GACGGGGAAGATGGGGATGG-3′ and 5′-CGGGTGGAACGTGGGGAA-3′), TET1 (5′-GTTTGGCTCCAAGGAAGGAA-3′ and 5′-GGAACAGGCTGAGTGAAACA), TET2 (5′-AGTTTCTGCCTCTTCCGTGG-3′ and 5′-TGGTTTTCTGCACCGCAATG-3′), TET3 (5′-TGCCAGGCCTTTATGACTTC-3′ and 5′-ACAAGTACCACACCGTTTCC-3′)and U1AsnRNP1 (5′-CAACGACAGCCGAGACATGTA-3′ and 5′-AGCCTCCATCAAATACCCATTC-3′) primer sets were designed using Primer Express (Applied Biosystems). Expression levels of the various transcripts were determined using the 2 − [*delta*][*delta]Ct method*. Values were normalized to expression of β-Actin mRNA, and represented as the mean value of three independent experiments performed in triplicate ± standard deviations.

### 450K DNA methylation arrays and analysis

RCC4-VHL, RCC4-mock, 786-mock, 786-VHL, U2OSshSCR, U2OSshVHL, MRC5s were grown in normoxia, hypoxia or treated with siDNMT1. Protein (method as previously described) was extracted from each sample and tested using western blot to validate presence of expected markers and ensure the experimental conditions were effective. Genomic DNA was extracted from the same samples using an extraction kit (Qiagen;) according to the manufacturer’s instructions. 1.5 µg of each sample was then bisulfite treated with a kit (EZ DNA methylation Kit; Zymo Research) according to manufacturer’s instructions. Bisufite treated samples were then loaded onto the Illumina 450 K array platform.

### Bioinformatics and statistical analysis

Processing and statistical analysis of the HumanMethylation450K array data was performed using the R statistical language version 3.2.2 and various packages of the Bioconductor project^33^. Specifically, minfi^34^ was used to load, apply SWAN normalization^35^, annotate probes and filter probes mapping to known SNPs. Differential methylation of loci between cell lines was tested using the Limma^36^ on the M-values^37^. Statistical significance for differential methylation was reached at 5% Benjamini-Hochberg false discovery rate and an absolute difference of M-values of 1. CpG loci were annotated as putative hypoxia-response elements (HREs) if they were part of the RCGTG motif.

Correlation between DNA methylation and gene expression was performed on differences of DNA methylation between tumor and normal of each patients and the differences of gene expression levels of the same samples using Pearson’s correlation. We compared differences in mRNA abundance of a given gene between patient-matched tumor and normal samples to the difference of DNA methylation levels of loci associated with that gene between the same specimens.

WGCNA package [PMID:19114008] was used to identify cluster of CpGs associated with the same gene that show similar changes of DNA methylation patterns. CpG clustering was only performed on genes which have at least five cis CpGs. The eigengene, which represents the weighted average value of methylation level of the cluster was extracted, and then a regression model was fit between each eigengene and expression of its associated gene.

## Electronic supplementary material

Supplementary Table 1

Supplementary Table 2

Supplementary Table 3

Supplementary Table 4

Supplementary Table 5

Supplementary Table 6

Supplementary Table 7

Supplementary Table 8
